# Longevity clinics: between promise and peril

**DOI:** 10.18632/aging.206330

**Published:** 2025-10-13

**Authors:** Marco Demaria

**Affiliations:** 1 European Research Institute for the Biology of Ageing (ERIBA), University Medical Center Groningen (UMCG), University of Groningen (RUG), Netherlands

**Keywords:** aging, longevity clinics, biomarkers, frailty, senescence

The idea of slowing, or even reversing, human aging has long occupied both science and imagination. While basic research over the past two decades has revealed hallmarks of aging and pointed toward possible interventions [1], the translation of these insights into accessible healthcare solutions remains in its infancy. Against this backdrop, longevity clinics, sometimes named age-management practices, personalized health centers, or wellness-longevity hybrids, have rapidly emerged across the globe. From USA to Switzerland, Singapore to Dubai, these clinics market comprehensive programs promising to monitor, manage, and mitigate biological aging.

At their core, longevity clinics claim to combine cutting-edge diagnostics with personalized interventions aimed at extending healthspan. A typical client may undergo genomic sequencing, multi-omics profiling, advanced imaging, full body scans, immune system assessments, microbiome analyses, and epigenetic testing. The results are then used to design individualized regimens that can include exercise prescriptions, nutritional guidance, nutraceuticals, sleep optimization, stress-management strategies, hormone replacement, or more experimental therapies such as stem-cell infusions, injection of peptides, plasma exchange and others. This approach gives a good example of what the medicine of the future should be: proactive, preventive, and fully personalized. However, some see it as a costly experiment bordering on pseudoscience.

The major issue is that longevity clinics not yet embedded within mainstream medical practice. They illustrate well both the enormous opportunities but also the very high risks inherent in translating geroscience into society. Understanding their potential, their limitations, and the conditions under which they might mature into credible engines of progress is crucial if we want the longevity movement to benefit populations.

Longevity clinics embody an important vision: healthcare is personalized, preventive, and engaged. They respond to a demand that traditional healthcare systems have failed to meet, which is optimization of healthspan. Several concepts should not be dismissed.

First, the potential to generate large-scale, longitudinal datasets on human aging. Unlike traditional clinical trials, which are highly controlled, time-limited, and often focused on specific diseases, longevity clinics engage individuals across years, sometimes decades, while capturing a broad range of biological and behavioral parameters. This deep phenotyping could reveal patterns of aging trajectories that are normally missed in conventional study designs currently undergoing in standard hospitals and clinics. Analysis and integration of collected datasets could highlight early biomarkers of decline, predictors of age-related disease, and identify subgroups that respond differently to interventions. With the help of artificial intelligence and machine learning data obtained could accelerate discovery and be potentially used to build actionable models.

Second, engagement of the patients. Clients are not passive recipients but are encouraged to track, monitor, and reflect on their own health metrics. This is exactly what we would like to achieve in geroscience: a cultural shift from reactive treatment to proactive management. Importantly, when individuals are deeply involved in understanding and managing their own biology, adherence improves, and lifestyle modifications, which remain the most robust interventions for healthy aging, become the norm.

Third, longevity clinics often act as early adopters of emerging diagnostics and interventions. Academic medicine is often constrained by excessive regulation and limited funding, and it may take years to validate and implement novel approaches. Private clinics operate more quickly and offer clients access to tools well before they are adopted in hospitals. When backed up by rigorous science, these tools and the analyses of the data obtained could shorten the translation from bench to bedside.

Yet for all their promise, most longevity clinics today fall far short of their potential. A major limitation is cost. Annual memberships frequently range from €10,000 to €50,000, with some “executive health packages” exceeding €100,000 (see Table 1). This makes them accessible only to wealthy elites, supporting a system of inequality in healthcare. Major issue is that the individuals most at risk of premature aging are normally coming from the lowest socio-economic levels.

**Table 1:** Examples of prominent longevity clinics, core offerings, and pricing.

**Clinic**	**Location(s)**	**Core offering**	**Typical cost**	**What’s included**
**Human Longevity Inc. (Health Nucleus)**	USA (San Diego, Scottsdale)	Whole-body MRI, coronary CT, genomics, multi-omics, concierge health plans	$8,000 (Executive Health) to $12,000+/yr (100+ plans)	Annual executive physicals, advanced imaging, genomics, physician oversight	
**Fountain Life**	USA (multiple), expanding globally	Tiered memberships (Core, APEX, EPIC) with MRI, cardiac CT, AI analytics, concierge	$10,500–$85,000/yr	Annual comprehensive imaging, labs, access to vetted therapies	
**Cenegenics**	USA (national network)	Hormone optimization, performance health, nutrition, fitness	$14,000–$21,000/yr	Initial eval ~$4,000, monthly physician visits, labs, HRT	
**Clinique La Prairie**	Switzerland (+ branches)	“Revitalisation” longevity weeks (diagnostics, therapies, spa)	CHF 20,950–48,520/week	Comprehensive testing, therapies, nutrition, luxury hospitality	
**Aviv Clinics**	Israel, UAE, USA (Florida)	12-week HBOT program with cognitive/physical training	$45,000–$60,000/program	60 HBOT sessions, imaging, cognitive tests, coaching	
**SHA Wellness**	Spain, Mexico, UAE	7–14-night “Advanced Longevity” programs	€9,500+ (program) + €600/night rooms	Diagnostics, nutrition, exercise, Eastern and Western therapies	
**Lanserhof**	Germany, Austria	Medi-detox longevity stays (diagnostics, FX-Mayr, therapies)	€6,600–8,600+ (program only)	Diagnostics, diet, exercise, cryotherapy; lodging extra	
**Palazzo Fiuggi**	Italy	Longevity full-body check-ups, diagnostics + hospitality	£10,299–11,949+ per stay	Advanced testing, therapies, nutrition, luxury resort stay	
**Chenot Palace Weggis**	Switzerland (+ other countries)	Chenot Method, detox + ageing-well programs	CHF 5,500–10,000+/week	7-night packages with diagnostics, strict diet, therapies	
**Echelon Health**	UK (Harley St, London)	One-day “Platinum” comprehensive check-up	£14,000/day	CTCA, MRI, colonography, ultrasound, bloods, specialist review	

The table highlights prominent and high-cost longevity clinics, but comes with several limitations that should be carefully considered: (1) profitability is not disclosed, only the program fees are public; (2) pricing is likely variable, as costs are often quoted as “from” rates; (3) the data capture is uneven, as medical clinic models (Human Longevity, Fountain Life) generate structured longitudinal data but resort programs (Clinique La Prairie, SHA, Chenot) are short-term and less standardized; (4) many clinics frame themselves as “wellness” rather than medical providers.

Beyond cost, there are also several issues with scientific rigor. It is not unusual for clinics to adopt unproven or risky therapies. Exotic supplements and intravenous cocktails are sold with minimal validation. Stem-cell infusions or experimental biologics are sometimes offered without robust safety data. In too many cases, commercial incentives overcome scientific rationale.

Interpretation of diagnostics is frequently problematic. Several tools used, such as biological age tests based on epigenetics or telomeres, are presented to clients as definitive scores, but their precision and clinical utility are still under debate. When multi-omics profiles are offered, they are often presented without a clear actionable meaning. The danger is that clients are overwhelmed by technology and data derived from it, but they eventually receive advice not fully scientifically supported.

The other major issue is that most clinics are pretty much disconnected from academic geroscience and from clinical geriatrics. This absence of collaboration undermines their ability to validate outcomes, to publish findings, or to contribute meaningfully to the field. This creates a vicious cycle where clinics are dismissed by scientists as pseudoscientific, and scientists are dismissed by clinics as too conservative or too critical.

Many clinics position themselves as wellness providers rather than medical facilities, so that they escape rigorous oversight. While this allows for some extra flexibility, it also enables practices that would not withstand the scrutiny of a clinical trial or hospital ethics board. The result is a grey zone where ambitious interventions can be marketed without adequate safety, accountability, or transparency.

All these come with the risk that the credibility of the entire field of longevity science is under threat. Claims of “reversing aging” or “guaranteeing 20 extra years of life” might attract customers but will surely underdeliver and invite skepticism.

Considering all the pros and cons, the question is how to make sure longevity clinics eventually support the field of geroscience.

First, integration with science is essential. Clinics should form partnerships with academics, clinicians, universities, research institutes, and hospitals. Data generated in clinics could be standardized, anonymized, and shared with academic consortia. Interventions could be structured as pragmatic clinical trials, with outcomes published in peer-reviewed journals. Scientists, in turn, could benefit from access to diverse datasets and motivated populations.

Second, harmonization of protocols is crucial. We need an agreement on metrics, biomarker panels, and reporting frameworks. Without standardization, data are not comparable and we lose an enormous opportunity.

Third, accessibility should be addressed. Some clinics will inevitably remain only for the wealthy. But alternative and parallel scalable models could bring core services to broader populations. Engagement with insurance companies and public health systems could speed up the democratization of healthy longevity. This will also help to have much more diversity in the datasets collected, potentially more effective in capturing the true heterogeneity of aging.

Fourth, we need more regulatory clarity. Operating in the wellness grey zone may be profitable, but it is unsustainable. Clear frameworks for what constitute medical versus wellness intervention, and for how safety and efficacy should be assessed, would benefit both clinics and clients. Clinics that voluntarily adopt higher standards of transparency and oversight will gain credibility.

Thus, longevity clinics represent both a warning and an opportunity. They warn us of the risks of commercialization overcoming science, of potential inequalities in providing access to healthcare and its lates technology, and of undermining credibility of the geroscience field. Yet they also offer a good example of a healthcare paradigm that society urgently needs based on a proactive, personalized, and preventive approach.

If they open themselves to integration, collaboration, and accountability, they could help accelerate the translation of geroscience into real-world benefits. On the other side, scientists, clinicians, policymakers, and entrepreneurs should engage constructively with this evolving sector to guarantee implementation and translation of rigorous science.

## References

[1] López-Otín C, et al. Cell. 2023; 186:243–78. 10.1016/j.cell.2022.11.001 36599349

